# My fellow

**Published:** 2009

**Authors:** Mahesh Pundlik Kate

**Affiliations:** Department of Neurology, Sri Chitra Tirunal Institute for Medical Sciences and Technology, Trivandrum, India. E-mail: maheshkate@yahoo.co.in

Today I stop your drugs,
I bid you good-bye,
The world is for you,
You have wings to fly.

But wait a minute and turn around,
Can you hear those similar seizing sounds?
They may be your kith or blood,
Or just another affected of this dread.

There is suffering, there are tears,
More than that, there is huge epilepsy fear.

In the darkness, you are the success light,
Wake them up, ask them to fight.

Drugs will help, education support,
It's time to act and build a rapport.

You have been blessed and so will be others,
Let's win their trust, stop their bothers.

Today I stop your drugs,
I bid you good-bye,
Let's join our hands,
So even others can fly.

Acknowledgment
My epilepsy patients.

